# Practical Tips for Construction of Custom Peptide Libraries and Affinity Selection by Using Commercially Available Phage Display Cloning Systems

**DOI:** 10.1155/2012/295719

**Published:** 2012-09-09

**Authors:** Keisuke Fukunaga, Masumi Taki

**Affiliations:** Bioscience and Technology Program, Department of Engineering Science, The Graduate School of Informatics and Engineering, The University of Electro-Communications (UEC), 7-5-1 Chofugaoka, Chofu, Tokyo 182-8585, Japan

## Abstract

Phage display technology is undoubtedly a powerful tool for affinity selection of target-specific peptide. Commercially available premade phage libraries allow us to take screening in the easiest way. On the other hand, construction of a custom phage library seems to be inaccessible, because several practical tips are absent in instructions. This paper focuses on what should be born in mind for beginners using commercially available cloning kits (Ph.D. with type 3 vector and T7Select systems for M13 and T7 phage, respectively). In the M13 system, Pro or a basic amino acid (especially, Arg) should be avoided at the N-terminus of peptide fused to gp3. In both systems, peptides containing odd number(s) of Cys should be designed with caution. Also, DNA sequencing of a constructed library before biopanning is highly recommended for finding unexpected bias.

## 1. Introduction

Phage display technology was born in 1985 when George Smith reported that foreign peptide could be displayed on the surface of filamentous bacteriophage [3]. Today, the phage display is a versatile tool for finding specific interactions between randomized library peptides/proteins on phage and target proteins, peptides, or other molecules. For example, it is applicable for generation of therapeutic peptides against cancer [4], microbe [5], novel functional protein [6], or fully humanized monoclonal antibody [7]. The advantages of the phage display technology over other selection methods are as follows. (1) Cost of a routine is cheap. (2) Time required for selection/amplification is fast. (3) Extreme care for handling, such as RNA isolation/selection, is not necessary. The phage is a DNA-containing virus that infects bacteria and makes many copies of the library within a very short time [8].

**Figure 1:**
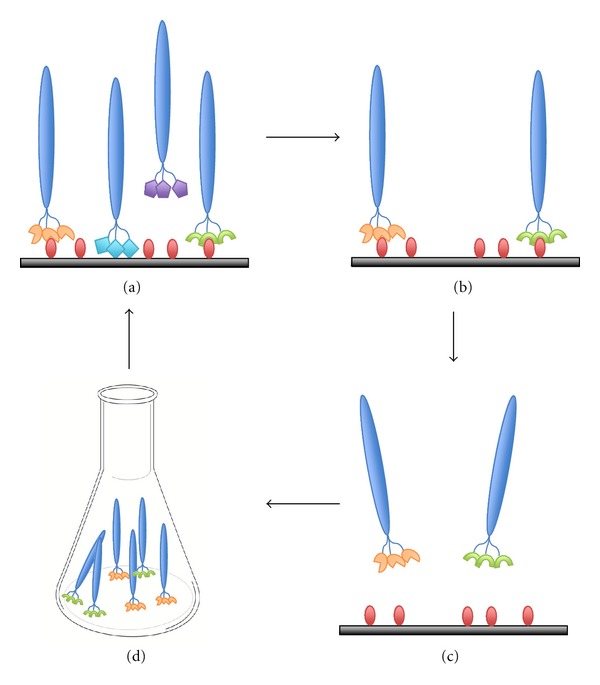
A typical procedure of the biopanning. (a) Incubation of phage library with an immobilized target. (b) Washing of unbound phage. (c) Elution of target-bound phage. (d) Amplification of the eluted phage for subsequent rounds of the biopanning.

A phage that specifically binds a target can be selected from mixtures of billions of phages, propagated by *in vivo* amplification, and then subjected to additional rounds of affinity selection (Figure 1). This whole process is so-called “biopanning” [9]. After multiple rounds of the biopanning, enrichment of target-binding phage can be assessed by phage titering and enzyme-linked immunosorbent assay (ELISA). Finally, the peptide displayed on the phage can be analyzed by DNA sequencing.

### 1.1. Categorization of Phage Display Systems

 Based on vector systems, the phage display systems can be categorized into two classes. One is a true phage vector system. The phage vector is often derived from genes encoding all phage proteins [10]. The library is to be cloned as a fusion with a component gene, which originally exists in the phage genome. Alternatively, some libraries are to be inserted in the same vector as an additional fusion gene encoding a displaying peptide and a phage protein [11]. 

Another is a phagemid vector system. The phagemid is a plasmid containing both a phage-derived replication origin and a plasmid-derived one [12]. A phage containing the phagemid can be generated only when phage components are secreted from bacterial host carrying a helper phage. In this system, two types of phages could be theoretically produced carrying either phagemid genome or helper-phage one. Practically, a helper phage with defective replication origin is used for the generation of phage proteins; production of the helper phage itself will be suppressed. This system yields a phage with the wild-type protein and library-fused one on the same virion, encoded by the helper phage and phagemid vector, respectively. Thus, numbers of the displaying peptides per virion from the phagemid system are less than those from the true vector system. This allows us to display not only small peptides but also large proteins [13], which is beyond the scope of this paper.

**Figure 2:**
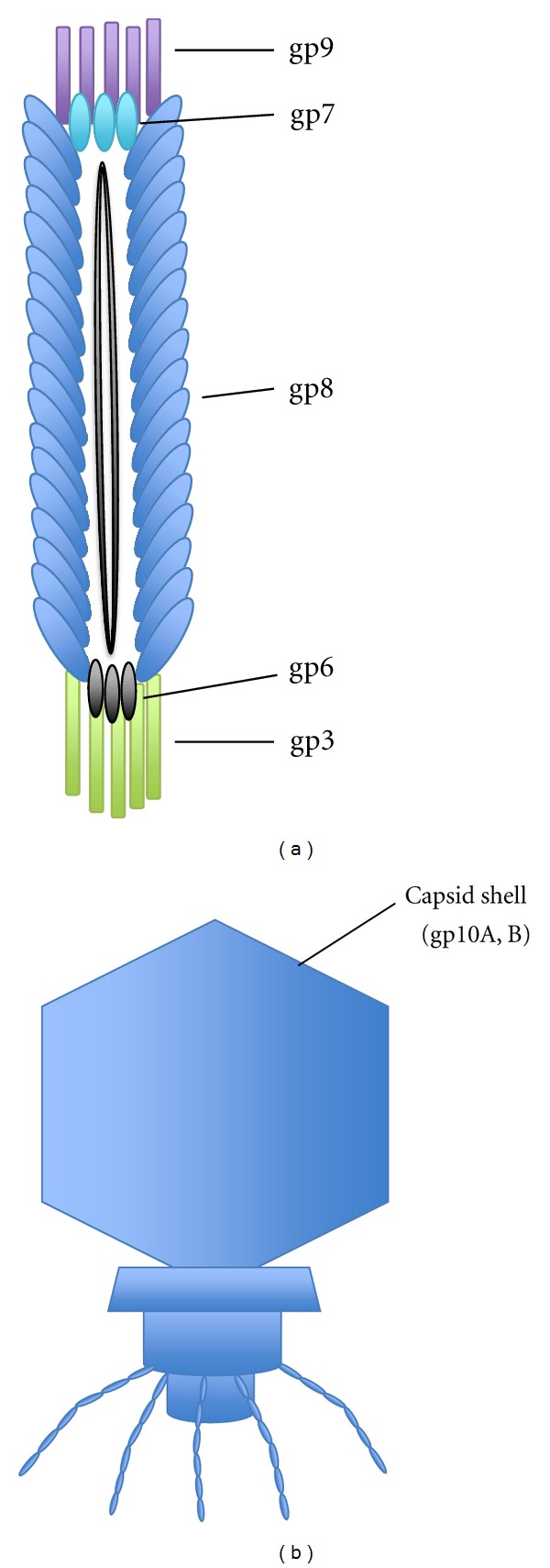
Structures of (a) a filamentous M13 bacteriophage and (b) a lytic T7 bacteriophage.

Among many different kinds of phages, M13 (filamentous bacteriophage) and T7 (lytic one) are exclusively used for the phage display. The M13 phage is composed of a circular single-stranded DNA genome and thousands copies of major capsid proteins (gp8) and capped by five copies of gp3 + gp6 on one end and five copies of gp7 + gp9 on the opposite (Figure 2). The most widely used M13 system is type 3. In this system, the peptide library is fused to the N-terminus of all five copies of the gp3. Other systems (e.g., type 33, type 8, etc.) are categorized by a peptide-displaying protein on the M13 phage and numbers of peptides per virion (Table 1) [14, 15].

The T7 phage is an icosahedral-shaped phage with a capsid shell that is composed of 415 copies of gp10, linear double-stranded DNA, and other proteins (Figure 2) [16]. The gp10 is made in two forms, gp10A (344 amino acids, aa) and its frameshifting product, gp10B (397 aa) [17]. In the T7 phage display systems, peptide library is always fused to the C-terminus of the gp10B. Numbers of peptides per virion and maximal size of the peptide are determined by the vector system (Table 1) [18].

### 1.2. Using Premade Phage Libraries

For screening, using a pre-made phage library is the most convenient way. Three types of M13 phage libraries, consisting of random linear/cyclic heptapeptides (Ph.D.-7/Ph.D.-C7C) and linear dodecapeptides (Ph.D.-12), are commercially distributable from New England Biolabs Inc. (NEB). In the C7C system, the randomized peptide is flanked by a pair of Cys, which are oxidized during the phage assembly to form an intramolecular disulfide bond. Several companies have constructed in-house pre-made peptide libraries; they provide screening services by using their phage libraries, instead of distributing ones. The chemical structures and features of the libraries are summarized in Table 2. Creative Biolabs Inc even accepts a service contract from a commercial pre-made library (e.g., Ph.D.-C7C system), a custom-constructed one in the company, or a hand-made one.

### 1.3. Construction of Custom Phage Library

Because of the limited kinds of resources, constructions of custom phage libraries are often performed by using kits available from NEB (Ph.D. Cloning System for M13 phage) or Merck Millipore (T7Select Cloning Kit for T7 one) [8]. Although these instructions are well described, several practical tips are missing in both of them, which may lead beginners to pitfalls such as obtaining severe inherent bias of amino acid sequence in the randomized region. This paper focuses on instant tips for the construction of peptide libraries and affinity selection by using the commercial resources. 

## 2. Ph.D. Cloning System

Ph.D. cloning system is based on a type 3 vector of M13 phage encoding N-terminal library peptide fused to a minor coat protein, gp3 [19]. Because gp3 plays a critical role for phage infection and randomized peptides are fused in all five copies of the gp3, infectivity of the M13 phage can be significantly affected by a sequence of the displaying peptide. Moreover, secretion of the M13 phage from *E. coli* closely depends on charges, hydrophilicity, and folding states of the displaying peptide [20, 21]. An amplification efficiency of the individual M13 phage clone is determined by a combination of the above infection and secretion rates. To avoid negative effects on the infection/secretion, one should be aware of the following in an insert DNA construction.

### 2.1. Signal Peptidase Cleavage 

Positively charged basic amino acids, Lys and Arg, near the signal peptidase cleavage site inhibit the secretion of phages [22]; the cationic residue blocks translocation across the inner membrane of *E. coli *[23]. If the N-terminus of the displaying peptide should be positively charged, Lys has to be evidently chosen; 6 out of 99 arbitrarily chosen clones of the commercial 12 mer library (Ph.D.-12) contained Lys at the terminus, whereas N-terminal Arg was never found in the same 99 clones [24]. If the N-terminal Arg is inevitable, using noncommercial prlA suppressor strains such as ARI180 or ARI182 may help to avoid the secY-dependent secretion problem [22].

Pro at the terminus is also cumbersome. When a Pro is located next to the cleavage site, it inhibits the signal peptidase cleavage [25, 26]. Only one N-terminal Pro out of the 99 clones was found in the Ph.D.-12 library [24].

If it is necessary to encode a specific amino acid sequence just after the signal peptidase cleavage site, prediction of the position-specific cleavage is recommended to avoid risks of inappropriate or insufficient cleavage. For example, an Internet server, SignalP [27], instantly does this, and we usually use 0.3 for the threshold D-cutoff value in the gram-negative bacteria mode.

**Figure 3:**
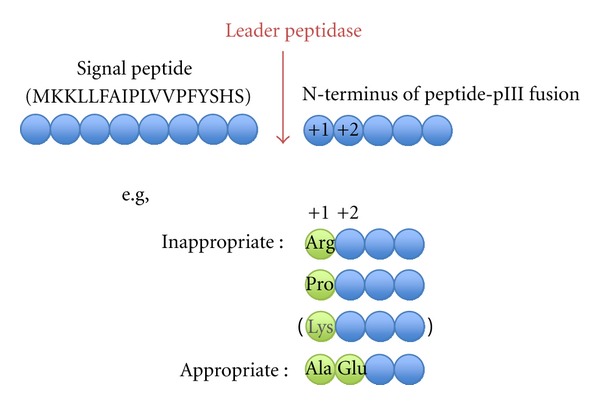
Sequence preference of the N-terminus of a peptide-pIII fusion in the M13 system.

If one does not have any favorites of particular N-terminal sequence just after the cleavage site, “Ala-Glu” or simple “Ala” should be the first choice. There is an overabundance of negatively charged amino acids (Glu and Asp) at +1 and +2 and Ala at +1, in gram-negative signal peptidase cleavage sites (Figure 3) [24].

### 2.2. Unpaired Cys in a Displaying Peptide 

If one generates a custom phage library displaying a disulfide-constrained peptide, an insert DNA encoding even number(s) of Cys, but not odd number(s), should be designed. This is because an intramolecular disulfide (S-S) bond could be formed between an unpaired Cys in a displaying peptide and an intrinsic Cys in the gp3 [28]. Phage assembly, infection, and/or secretion could be prevented by this unfavorable disulfide bond [24, 29]. It has been stated that an almost complete absence of odd number(s) of Cys was observed in the displaying peptide [28, 30], which is also identical to our experience. For example, when we sequenced 10 independent M13 phage clones encoding Cys-X_7_-Cys where the X stands for any randomized amino acid, no Cys was observed in the X_7_ region; only the designated Cys at both ends seemed to form an intramolecular disulfide bond (unpublished results). Given the difficulty, if one still tries to generate a phage library containing odd number(s) of Cys, M13 phages constructed by disulfide-free gp3 [1, 31] might be useful without using the Ph.D. system.

## 3. T7Select Cloning System

Unlike the filamentous M13 system, T7 capsid shell displaying peptide library is not involved in phage infection and/or secretion. Indeed, it has been proven that libraries of the T7 phages exhibit less sequence bias than those of the M13 ones [29]. This is a great advantage for library construction, because it is less necessary to pay attention to the amino acid sequences described above. The T7 system is also good at displaying a rigid motif with a hydrophobic domain, namely, Trp cage [32]. This peptide motif was never displayed on the M13 system, presumably because the hydrophobic domain was anchored to the inner membrane of the *E. coli *prior to the phage assembly [32].

### 3.1. Codon Usage

 To the best of our knowledge, there is no description of a relationship between codon usage and bias against translation for the T7 system in *E. coli*; in the M13KE system, it is reported that rare codons of *E. coli* seldom affect the bias of peptide libraries [24]. To avoid potential risks that minor codons could stress the translation system [33, 34], we simply use major codons (Table 3) for a nonrandomized region of a synthetic DNA insert.

### 3.2. Unpaired Cys in a Displaying Peptide 

**Figure 4:**
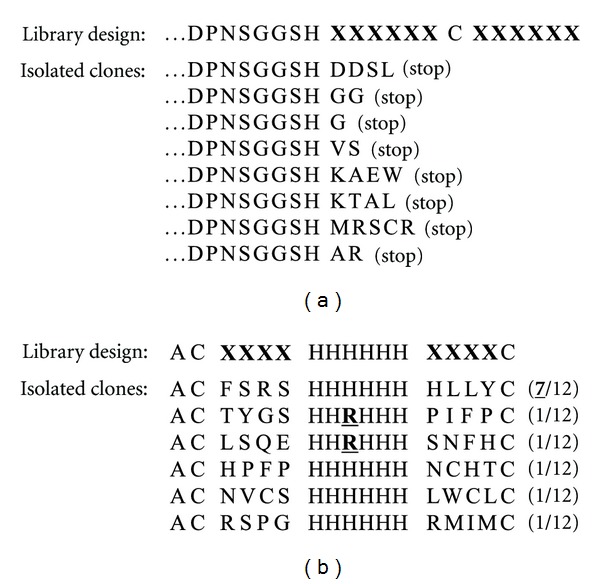
Unexpectedly isolated clones with high bias after library constructions. Bold “X” indicates any amino acids. (a) Stop codon appearance before the designated Cys. A combination of T7Select 415 vector and *E. coli* BL21 strain was used for *in vitro* packaging of the T7 phage, and individual clones were subjected to DNA sequencing. (b) Enrichment of a specific sequence and a mutation of the designated His (underlined). Randomly selected 12 individual clones of the M13 phage library were subjected to DNA sequencing. Parentheses indicate numbers of obtained clones.

In our experiment, when a T7Select415-1b vector was used for the T7 packaging, the T7 phage failed to display a designated unpaired Cys (unpublished results). In this case, the library insert DNA was constructed using the genetic code of (NNK)_6_-TGC-(NNK)_6_, which encodes X_6_-Cys-X_6_. DNA sequencing of 8 independent phage clones revealed that peptides were truncated by the appearance of a TAG stop codon before the designated Cys that was supposed to be translated (Figure 4(a)).

The capsid shell used for randomized peptide display is composed of 415 copies of gp10 [35]. A structural study of T7 procapsid shell suggested that the gp10 might play an important role in the interaction between capsid shell and scaffolding proteins [36]. The designated Cys in the library peptide fused to the gp10 might form an intermolecular disulfide bond with the same kind of unpaired Cys in a neighboring library peptide. It also might form an intramolecular one with an intrinsic Cys in the gp10. Too many unpaired Cys may inhibit proper/efficient assembly of the capsid shell proteins. Although we do not have direct evidence for this hypothesis, Rosenberg et al. also speculated that some peptide sequences might be unfavorable for the T7Select415 system [18].

### 3.3. Paired Cys in a Displaying Peptide 

Phages displaying the cyclic peptide by an intramolecular disulfide bond tend to exhibit higher target-binding ability, because their rigid structures minimize conformational entropy loss associated with the binding [37, 38]. Therefore, this kind of phage library is dominantly used for screening on the basis of not only M13 systems (e.g., Ph.D.-C7C library from NEB [37, 39]) but also T7 ones [40, 41]. Disulfide constrained library of the T7 phage is most frequently constructed by using T7Select10-3b [29, 42] or 415-1b vector [2, 43–45] (Table 2).

For generation of the disulfide constrained (S-S) library using the T7Select415 system, it is recommended in the manual (Merck Millipore) to use *E. coli* Origami B or Rosetta-gami B strains, which tends to enhance disulfide bond formation in the cytoplasm. However, these strains may not be required for the library constructions. By using *E. coli* BLT5615 strain included in the T7 kit with the T7Select10-3b [29] or 415-1b [2] vector, the constrained library peptides were successfully displayed on the T7 phage, and high-affinity cyclic peptides were obtained.

### 3.4. Features of the T7 System 

One of the features of the T7 phage, which grows much faster than the M13 one, is that it decreases the time for phage titering and amplification. After infection, clear plaques of T7 phages will usually appear within 2-3 hours on LB plate with no additives. Liquid amplification of the T7 phage after affinity selection can also be conducted within the same time. 

It is also attractive for beginners that the T7 system does not require any special instruments like an electroporator for the library construction. Contrary to the kit instructions, ultracentrifugation of the T7 phage with CsCl is not necessary for all purification processes of ELISA assay and DNA sequencing. General procedure using polyethylene glycol (PEG)/NaCl with a conventional rotator is enough for the T7 phage purification, in the same way as the M13 system.

The T7 system can be useful for direct recovery of the highest-affinity phage with a very slow off-rate from a target-linked solid support. It has been reported that a target-bound lambda phage can be directly amplified by the addition of *E. coli* in midlog phase [46]. In a similar way, a library peptide displayed on the capsid shell does not interfere with the infectivity of the T7 phage. Indeed, we have experienced that a streptavidin-binding peptide containing the consensus sequence (HPQ [47]) was successfully obtained by this direct method (unpublished results). In the M13 system, phages may also be eluted by the addition of the host bacterial cells; however the elution of the highest-affinity binders may be hindered.

A minor drawback of the T7 system is that it is relatively expensive to construct a library with a high diversity. In a typical case, six whole tubes of T7 packaging extracts in a T7Select packaging kit (ca. $410) are required to obtain a diversity of 4.1 × 10^8^ pfu [2].

### 3.5. Handling Precautions

 It should be emphasized that *in vitro* packaging has to be performed with extreme care. One must keep a stringent condition of the temperature and mixing. Only “fresh” T7 packaging extract will make a high quality library; freezing and thawing of the extract will result in apparent reduction of the packaging efficiency.

Diluted T7 phages with a buffer or water tend not to be infective. It should be diluted with a buffer containing a protectant such as gelatin or a growth media such as TB or LB.

## 4. Importance of DNA Sequencing for Finding Unfavorable Bias and False Positives at an Early Stage of the Affinity Selection

After the electroporation for the Ph.D. system or the packaging for the T7Select system, a qualitative assessment of the phage library should be performed by DNA sequencing prior to the biopanning. We always confirm it by a conventional DNA sequencer with at least 10 independent phage clones. For example, we obtained highly biased sequences when the random library encodes constraints with a His_6_-tag (Ala-Cys-X_4_-His_6_-X_4_-Cys) (unpublished results; Figure 4(b)). In this case, a specific sequence was predominantly enriched (7 out of 12 arbitrarily chosen clones). In addition, one of the designated His at the 3rd position of the His_6_-tag was mutated to Arg accompanied with a codon replacement from CAC to CAT. Nature seems to exclude the constrained His-tag in the M13 system, and such a library should not be used for the biopanning.

### 4.1. Advantage of High-Throughput DNA Sequencing 

**Figure 5:**
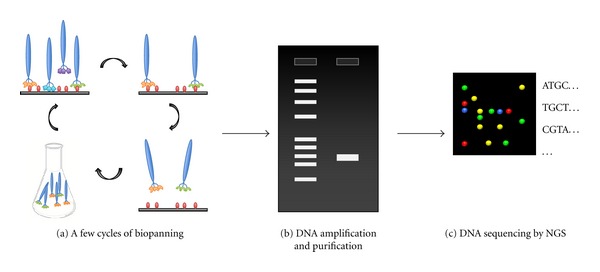
Phage display screening with next-generation sequencing. (a) Biopanning with one or two cycle(s). (b) Randomized region of phage DNA is amplified with Polymerase Chain Reaction (PCR). The products are subjected to gel electrophoresis followed by further DNA purification. (c) Purified DNA is analyzed by a next-generation sequencer.

A next-generation sequencer (NGS) makes it possible to sequence millions of inserts in parallel. If the NGS is available, one million reads of the library clones would be ideal for finding target-binding sequences even after first round of the biopanning (Figure 5) [48]. If false positive sequences such as target-unrelated (e.g., plastic or BSA) binders or propagation accelerating peptide (e.g., HAIYPRH [49]) are predominantly enriched at an early stage, further biopanning will be useless. These meaningless false-positive sequences are well described and summarized in a recently published review [50] and can be found easily with online databases (SAROTUP [51], http://immunet.cn/sarotup/; PepBank [52], http://pepbank.mgh.harvard.edu/). Once candidate clones are selected after several rounds of biopanning, the false-positive sequences should be excluded in the same manner.

### 4.2. Precautions for Conventional DNA Sequencing 

If the DNA sequencing is performed by a conventional sequencer but not by the NGS, one should be aware that the DNA sequencing of 50 randomly chosen clones after first or second rounds of the biopanning would be completely uninformative for finding target binders, because the population will be lacking [48]; it should be performed at a later round.

## 5. Conclusions

We summarized merits and demerits of the M13 and T7 systems in Table 4. It seems the T7 system is easier to handle for beginners, because there are several engineering tolerances in it. Additionally, the T7 phage is stable to detergents and denaturants, such as 1% sodium dodecyl sulfate (SDS), urea (up to 4M), and guanidine-HCl (up to 2M), for eliminating nonspecific binders during the biopanning. Although the T7 phage is robust against not only the chemicals but also an alkaline condition (pH 10), it is fragile at acidic conditions below pH 4. If an elution from target-linked solid support under the lower pH is necessary, the M13 system should be the first choice.

In both systems, the DNA sequencing of a constructed phage library before biopanning is highly recommended for finding unexpected bias.

## Figures and Tables

**Table 1:** Features of various systems of M13 and T7 phages.

	System	Size limit	Numbers of peptides per virion	Presentation region
M13	3	Unknown	5	N-terminus to gp3
	33	No limit	<1	
	3 + 3			
	8	Short	>2,700	N-terminus to gp8
	88	Unknown	<300	
	8 + 8	No limit	100–1000	
	8 + 8	Unknown		C-terminus to gp8
	6 + 6	No limit	<1	N-terminus to gp6
	6 + 6			C-terminus to gp6
	9 + 9			N-terminus to gp9

T7	T7Select1-1	1200aa	<1	C-terminus to gp10B
	T7Select1-2	900aa		
	T7Select10-3	1200aa	5–15	
	T7Select415-1	50aa	415	

**Table 2:** Consignment services of phage display with in-house libraries.

Company name	Peptide design	Peptide structure
Creative Biolabs	X_10_, X_16_, or X_20_^∗1^	linear
Dyax	X_a_CX_b_CX_c_^∗1^	cyclic
Bicycle Therapeutics	X_a_CX_b_CX_c_CX_d_^∗2^	cyclic with a non-natural linker

X stands for any randomized amino acid.

^∗1^The library was built by varying 19 aa at the randomized positions; the codon encoding Cys is excluded.

^∗2^Bicyclic peptide library was made via thioether linkages [1].

**Table 3:** Codon usage in *E. coli* K-12 strain.

Amino acid	Codon	Codon frequency (%)
Phe	UUU	1.97
	UUC	1.50

Leu	UUA	1.52
	UUG	1.19
	CUU	1.19
	CUC	1.05
	CUA	0.53
	CUG	4.69

**Ile**	AUU	3.05
	AUC	1.82
	**AUA**	**0.37**

Met	AUG	2.48

Val	GUU	1.68
	GUC	1.17
	GUA	1.15
	GUG	2.64

Ser	UCU	0.57
	UCC	0.55
	UCA	0.78
	UCG	0.80
	AGU	0.72
	AGC	1.66

Pro	CCU	0.84
	CCC	0.64
	CCA	0.66
	CCG	2.67

Thr	ACU	0.80
	ACC	2.28
	ACA	0.64
	ACG	1.15

Ala	GCU	1.07
	GCC	3.16
	GCA	2.11
	GCG	3.85

Tyr	UAU	1.68
	UAC	1.46

His	CAU	1.58
	CAC	1.31

Gln	CAA	1.21
	CAG	2.77

Asn	AAU	2.19
	AAC	2.44

Lys	AAA	3.32
	AAG	1.21

Asp	GAU	3.79
	GAC	2.05

Glu	GAA	4.37
	GAG	1.84

Cys	UGU	0.59
	UGC	0.80

Stop	UAA	0.18
	UAG	0.00
	UGA	0.10

Trp	UGG	1.07

**Arg**	CGU	2.11
	CGC	2.60
	**CGA**	**0.43**
	**CGG**	**0.41**
	**AGA**	**0.14**
	**AGG**	**0.16**

Gly	GGU	2.13
	GGC	3.34
	GGA	0.92
	GGG	0.86

Codon frequency (%) is defined as the percent frequency of each codon which matches in whole open-reading frame of the *E. coli* K-12 genome. Minor codons (bold letters; below 0.5%) could be avoided for insert DNA construction. This table was cited from codon usage database (http://www.kazusa.or.jp/codon/) with some modifications.

**Table 4:** Comparison of the M13 and T7 phage in library construction and affinity selection.

	M13 phage	T7 Phage
Cost	Routinely cheap, requires	Routinely expensive, no additional
	electroporator and cuvettes	instruments required
Site of library peptide	N-terminus of gp3	C-terminus of gp10B
Library size (per *μ*g DNA)	~10^9^	~10^8^ [2]
Peptide sequence bias	Highly biased	Less biased
Time required for phage tittering/amplification	Long	Short
